# NREM Sleep Instability in Pediatric Migraine Without Aura

**DOI:** 10.3389/fneur.2019.00932

**Published:** 2019-08-27

**Authors:** Michele Roccella, Rosa Marotta, Francesca Felicia Operto, Daniela Smirni, Francesco Precenzano, Ilaria Bitetti, Giovanni Messina, Francesco Sessa, Giulio Di Mizio, Carla Loreto, Monica Salerno, Vincenzo Russo, Paolo Murabito, Beatrice Gallai, Maria Esposito, Diego Iacono, Marco Carotenuto

**Affiliations:** ^1^Department of Psychology, Educational Science and Human Movement, University of Palermo, Palermo, Italy; ^2^Department of Medical and Surgical Sciences, University “Magna Graecia”, Catanzaro, Italy; ^3^Basic Medical Sciences, Neuroscience and Sense Organs Department, University of the Study of Bari “Aldo Moro”, Azienda Ospedaliero-Universitaria Consorziale Policlinico di Bari, Bari, Italy; ^4^Clinic of Child and Adolescent Neuropsychiatry, Department of Mental Health, Physical and Preventive Medicine, University of Campania “Luigi Vanvitelli”, Naples, Italy; ^5^Department of Clinical and Experimental Medicine, University of Foggia, Foggia, Italy; ^6^Department of Legal, Historical, Economic and Social Sciences, University of Catanzaro, Catanzaro, Italy; ^7^Department of Biomedical and Biotechnological Sciences, University of Catania, Catania, Italy; ^8^Department of Medical, Surgical and Advanced Technologies “G.F. Ingrassia”, University of Catania, Catania, Italy; ^9^Institute of Ophthalmology, University of Foggia, Foggia, Italy; ^10^Department of Clinical and Experimental Medicine, University of Catania, Catania, Italy; ^11^Department of Surgical and Biomedical Sciences, University of Perugia, Perugia, Italy; ^12^Neurodevelopment Research Lab, Biomedical Research Institute of New Jersey, Cedar Knolls, NJ, United States; ^13^Neuroscience Research, MidAtlantic Neonatology Associates, Atlantic Health System, Morristown, NJ, United States; ^14^Neuropathology Research, MANA/Biomedical Research Institute of New Jersey, Cedar Knolls, NJ, United States

**Keywords:** migraine without aura (MoA), NREM sleep instability, cyclic alternating pattern (CAP) analysis, sleep macrostructure, full overnight polysomnography

## Abstract

Children with migraine headaches appear to have a range of sleep disturbances. The aim of the present study was to assess the NREM sleep instability in a population of school-aged individuals affected by migraine without aura (MoA). Thirty-three children with MoA (20 males, 13 females, mean age 10.45 ± 2.06 years) underwent to overnight Polysomnographic (PSG) recordings and Cyclic Alternating Pattern (CAP) analyses accordingly with international criteria. MoA group showed a reduction in sleep duration parameters (TIB, SPT, TST; *p* ≤ 0.001 for all) and in arousal index during REM sleep and an increase in awakenings per hour (AWK/h) vs. Controls (C) (*p* = 0.008). In particular, MoA children showed a reduced CAP rate% (*p* ≤ 0.001), CAP rate% in S1 (*p* ≤ 0.001) and CAP rate% in SWS (*p* = 0.004) vs. C. Moreover, A phases distribution were characterized by a reduction in slow wave components (total number CAP A1%, CAP A1 index) (*p* ≤ 0.001) and an increase of fast components representation (total number of CAP A2% and CAP A3%) (*p* < 0.001) in MoA vs. C. Moreover, MoA children showed an increased A1 and A2 mean duration (*p* ≤ 0.001). Our findings show a reduction of arousability in MoA group and lower NREM lower sleep instability associated with MoA in children.

## Introduction

Sleep and headache are widely related from a clinical point of view. The biological relationship between sleep and pain processing is not fully understood yet. Presently, a unique hypothesis about the mutual inter-relationship between sleep and primary headaches cannot be presented. In this picture, we can assume that various mechanisms may be responsible for the different clinical features observed in association with headache and sleep (1). However, the connection between sleep disorders and primary headaches is clinically relevant since both conditions tend to establish mutual interrelationships that influence each other (2–4). In this context, the clinical observation raises questions regarding the pathogenesis of these disorders, involving pivotal cerebral structures (i.e., thalamus, hypothalamus, and some brainstem nuclei) and specific neurochemical pathways both in pain perception and sleep regulation.

The hypothalamus is crucial in both headache pathogenesis and sleep-wake cycle regulation because of its connection with the anti-nociceptive system [i.e., medulla oblongata, serotoninergic raphe nucleus, noradrenergic locus coeruleus, and periaqueductal gray matter (PAG)] the stimulation mediated by orexin on ventro-lateral part of PAG, and the inhibition on the anti-nociceptive activity in the caudal trigeminal nucleus (5–7).

In childhood, the most frequent primary headaches could be considered the migraine without aura (MoA) and tension-type headache with a prevalence of 2–17 and 0.9–24%, respectively (8, 9). Children with migraine headaches appear to have more frequent sleep troubles consisting in insufficient sleep, maternal co-sleeping, longer sleep latency, more bedtime resistance, shorter sleep duration, daytime sleepiness, night awakenings, sleep anxiety, parasomnias, and sleep-disordered breathing compared to children from a normative community sample (10).

To date, a limited number of polysomnographic studies carried out on patients with migraine, with no conclusive association about any peculiar characteristics of sleep architecture, although migraine attacks seem to be linked to REM stages and associated with a large amount of deep sleep (11). Moreover, Goder et al. (12) reported that migraine attacks were preceded by a significant decrease in arousals number, REM density, and in beta power band in the slow wave sleep, and by a decrease in alpha power during the first REM period. However, Vendrame et al. evidenced a high prevalence of sleep fragmentation (i.e., sleep disordered breathing, high rate of awakenings) in children with mild or severe migraine with an increasing related to the severity of symptoms (13, 14). In a PSG study Karthik et al. showed significantly lower sleep efficiency, prolonged sleep onset latency, lesser stage 4 and NREM sleep, and a greater number of total awakenings in migraineurs compared to the controls (15). In 2016, Nayak et al. showed a decreased REM arousability as well as a decreased overall CAP rate and CAP cycling in adult patients with migraine as compared to controls (16). In this perspective, we have hypothesized that the sleep parameters (such as macrostructure and microstructure) could be different in children affected by MoA respect of typical developing healthy comparisons (control subjects [C]). Therefore, the aim of the present study was to assess the NREM sleep instability in a population of school- aged individuals affected by MoA vs. C.

## Materials and Methods

### Study Population

Thirty-three children affected by migraine without aura (MoA) (20 males, 13 females, mean age 10.4 ± 2.0 years) underwent to an overnight PSG recording, after one adaptation night to avoid the first-night effect in the Sleep Laboratory of Child and Adolescent Neuropsychiatry at the Università degli Studi della Campania “Luigi Vanvitelli”, Campania Region, Italy. The diagnosis of migraine was made according to international criteria (17). None of those recruited children had taken prophylactic medication or neither any other regular medication for at least the 2 weeks prior to neither recruitment nor migraine attacks for 48 h at least before the study began.

Following recruitment, to verify the headache characteristics monthly headache frequency and mean headache duration was assessed from daily headache diaries kept by all the children. The headache intensity was assessed on a visual analog (VAS) scale. Exclusion criteria were neurological (i.e., epilepsy, neuromuscular disorders) or psychiatric symptoms (Attention Deficit Hyperactivity Disorder, anxiety, depression, behavior problems), mental retardation (IQ ≤ 70), borderline intellectual functioning (IQ ranging from 71 to 84), and referred signs suggestive for the presence of sleep-related breathing disorders (i.e., habitual snoring, nocturnal apneas), for periodic limb movement disorder (i.e., nocturnal hyperkinesias) and recurrent parasomnias (>3 episodes per week).

In order to compare the data from MoA children with a control group, 52 healthy children (C) (29 males, 23 females, mean age 9.9 ± 2.4 years) were enrolled from the Campania and Sicily regions schools. The subjects of both groups were recruited from the same urban area, were all of Caucasian origin and had middle socioeconomic status.

The investigation was carried out in accordance with the principles of the Declaration of Helsinki (18). All adult subjects provided written informed consent and a parent or guardian of any child participant provided written informed consent on their behalf. All procedures were performed in accordance with International guidelines and were approved by Scientific Committee of University of Palermo (n° 2015-001160-19).

### Polysomnographic Evaluation (PSG)

Full overnight PSG recordings were performed according to international criteria (19–21), started at the subject's usual bedtime and continued until spontaneous morning awakening. The PSG scoring was visually analyzed by means of Hypnolab 1.2 sleep software analysis (SWS Soft, Italy) and the following conventional sleep parameters were evaluated:

1. Time in bed (TIB);
2. Sleep period time (SPT);
3. Total sleep time (TST);
4. Sleep latency (SL);
5. First REM latency (FRL);
6. Number of stage shifts/hour (SS/h); Number of awakenings/hour (AWN/h);
7. Sleep efficiency (SE%);
8. Percentage of SPT spent in wakefulness after sleep onset (WASO%);
9. Percentage of SPT spent in sleep stages 1 (N1%), 2 (N2%), slow-wave sleep (N3%), and REM sleep (REM%).

Moreover, the Arousal Index during the REM sleep was calculated.

About respiratory parameters, central, obstructive and hypopnea events were counted according to the standard criteria (22) considering as abnormal an Apnea/Hypopnea index (AHI) >1 (23). Moreover, periodic limb movements (PLMs) events were identified (24) and a PLMI≥5 was considered abnormal.

### Cyclic Alternating Pattern (CAP) Analysis

CAP was scored following the standard criteria defined by Terzano et al. (25). CAP A phases have been subdivided into a 3-stage hierarchy of arousal strength: A1 is defined as the A phase with synchronized EEG patterns (intermittent alpha rhythm in stage 1 and sequences of K complexes or delta bursts in the other NREM stages) associated with mild or trivial polygraphic variations; A2 is defined as the A phase with desynchronized EEG patterns preceded by or mixed with slow high-voltage waves (K complexes with alpha and beta activities, K alpha, and arousals with slow-wave synchronization) linked to a moderate increase of muscle tone and/or cardiorespiratory rate; and A3 as the A phase with desynchronized EEG patterns alone (transient activation phases or arousals) or exceeding two thirds of the phase A length and coupled with a remarkable enhancement of muscle tone and/or cardiorespiratory rate (25).

The following CAP parameters were measured:

- CAP time (temporal sum of all CAP sequences) in NREM sleep;
- The CAP rate (percentage of total NREM sleep time occupied by CAP sequences);
- The number and duration of CAP cycles; the number and duration of CAP sequences;
- The number, duration, and percentage of A phases (including the phase A subtypes);
- A1 index (number of A1 phases per hour of NREM sleep);
- A2 index (number of A2 phases per hour of NREM sleep);
- A3 index (number of A3 phases per hour of NREM sleep);
- and the number and duration of B phases.

### Statistical Analysis

The comparisons between sleep architecture and CAP parameters, obtained in MoA children and typically developing children (C), were carried out by the Mann–Whitney *U* test. Bonferroni correction was applied. *P*-values <0.01 were considered statistically significant. STATISTICA (data analysis software system), version 6, StatSoft, Inc. (2001) was used for all statistical tests.

## Results

The two groups (MoA and C) were matched for age, sex, and z-score Body Mass Index (z- BMI) (Table 1). The migraine characteristics such as frequency, intensity and duration of attacks were showed in Table 1. None of the children with migraine in our series were affected by a migraine attack during the sleep study.

**Table 1:** The comparison between migraine without aura (MoA) and typically developing children (Control) groups in age, sex distribution, and *z*-score Body Mass Index (z-BMI).

	**MoA**	**Control**	***p***
	**(*N* = 33)**	**(*N* = 52)**	
Age	10.45 ± 2.06	9.98 ± 2.42	0.355
Sex (M/F)	20/13	29/23	0.830
z-BMI	0.43 ± 0.51	0.59 ± 0.41	0.115
Frequency (attacks/month)	9.18 ± 2.84	–	–
Duration (h)	6.39 ± 2.56	–	–
Intensity (VAS)	7.45 ± 2.39	–	–

*The frequency, duration, and intensity (assessed with Visuo-analogic Scale, VAS) of migraine attacks were described for MoA group. The t-Test and the Chi-square test, when appropriated, were applied. p values <0.05 were considered statistically significant*.

As for the macrostructural findings, MoA group showed significant reduction in sleep duration parameters (TIB, SPT, TST; *p* ≤ 0.001 for all) and a significant increase in awakenings per hours (AWK/h) vs. C (*p* = 0.008; Table 2). Moreover, the Arousal Index during REM sleep was lower in MoA vs. C children (*p* < 0.001; Table 2).

**Table 2:** Polysomnographic sleep macrostructure findings in MoA and normal control group.

	**MoA control**			**Mann-Whitney** ***U*****-Test**		
	***N* = 33**		***N* = 52**			
	**Mean**	**Std.Dev**.	**Mean**	**Std.Dev**.	***U***	p*
TIB-min	473.697	46.459	588.933	88.132	185.0000	0.000
SPT-min	446.076	47.033	557.462	83.305	186.0000	0.000
TST-min	397.712	70.858	529.356	78.345	159.0000	0.000
SOL-min	15.470	13.070	21.288	17.928	658.5000	NS
FRL-min	143.727	58.193	124.423	50.707	695.0000	NS
SS-h	9.242	2.700	8.756	3.490	787.5000	NS
AWN-h	4.088	2.615	2.137	1.826	472.5000	0.008
SE%	83.888	12.056	90.058	5.205	647.5000	NS
WASO%	11.058	11.485	4.910	4.303	620.5000	NS
N1%	2.333	2.266	2.800	3.543	843.0000	NS
N2%	36.200	9.286	43.269	24.558	667.0000	NS
N3%	34.015	10.684	31.194	9.466	759.0000	NS
REM%	16.361	7.217	21.200	5.338	526.0000	NS
AHI	0.561	0.240	0.658	0.211	649.0000	NS
ODI	0.592	0.174	0.587	0.148	834.5000	NS
PLM%	2.970	1.088	2.748	0.889	769.5000	NS
REM arousal index	6.606	2.164	13.269	2.657	44.5000	0.000

*Differences evaluated with the Mann-Whitney-U test, among children affected by migraine without aura (MoA) and control group in the following parameters: TIB, Time in bed (in minutes); SPT, Sleep period time (in minutes); TST, Total sleep time (in minutes); SOL, Sleep onset latency (in minutes); SS/h, Stage shifts per hour; AWN/h, Awakenings per hour; SE%, Sleep efficiency percentage; WASO% percentage, Wakefulness after sleep onset percentage; S1 and S2%, Sleep stages N1 and N2 percentages; N3%, Slow-wave sleep percentage; REM%, Rapid eye movement sleep percentage; AHI, Apnea/Hypopnea Index; ODI, Oxygen Desaturation Index; PLM, Periodic Limb Movements; R arousal Index (REM arousal index). NS means not significant. p values <0.01 were considered as significant*.

* *Bonferroni-corrected value*.

As for the NREM sleep analysis, MoA children showed a reducing in CAP rate% (*p* ≤ 0.001), CAP rate% in N1 (*p* ≤ 0.001) and in CAP rate% in SWS (*p* = 0.004) vs. C. Moreover, the A phases distribution were characterized by significant reduction in slow wave components (Total number CAP A1%, CAP A1 index) (*p* ≤ 0.001) and an increasing in fast components representation (Total number of CAP A2% and CAP A3%) in comparison to C. MoA children show also an increased A1 and A2 mean duration (*p* ≤ 0.001; Table 3) in comparison to the healthy control group (C).

**Table 3:** The mean differences, evaluated with the Mann-Whitney-*U* test, among children affected by migraine without aura (MoA) and control group in the following parameters: CAP refers to cyclic alternating pattern; CAP rate (percentage of total NREM sleep time occupied by CAP sequences); percentage and duration of each A phase subtype; A1 index (number of phases A1 per hour of NREM sleep, and of N1, N2, and N3 sleep stage); A2 index (number of phases A2 per hour of NREM sleep, and of N1, N2, and N3 sleep stage); A3 index (number of phases A3 per hour of NREM sleep, and of N1, N2, and N3 sleep stage); duration of B phases; number and duration of CAP sequences.

	**MoA**		**Control**		**Mann-Whitney**	***U*-Test**
	***N* = 33**		***N* = 52**			
	**Mean**	**Std.Dev**.	**Mean**	**Std.Dev**.	***U***	p*
CAP_Rate%	26.539	12.506	34.346	6.496	423.0000	0.001
CAP_Rate%N1	5.817	18.501	19.877	18.025	256.0000	0.000
CAP_Rate%N2	21.197	11.414	28.288	10.519	521.5000	NS
CAP_Rate%N3	35.785	18.298	47.548	7.043	452.0000	0.004
Tot_num_A1%	47.224	21.804	79.233	11.871	135.5000	0.000
Tot_num_A2%	36.612	19.302	13.087	12.124	154.0000	0.000
Tot_num_A3%	16.594	9.725	7.683	3.236	287.0000	0.000
A1_mean_dur	13.600	4.463	5.312	1.632	39.0000	0.000
A2_mean_dur	18.079	6.269	8.923	2.632	259.5000	0.000
A3_mean_dur	13.342	3.714	15.981	6.598	684.5000	NS
A1_index	14.776	9.607	40.779	10.005	50.0000	0.000
A2_index	11.521	10.327	7.063	6.324	607.5000	NS
A3_index	4.288	3.811	3.535	2.943	739.0000	NS
B_mean_dur	22.715	4.412	22.237	4.066	784.0000	NS
Cycle_mean_dur	38.024	6.347	28.673	4.881	245.0000	0.000
Seq_mean_dur	211.852	82.054	202.685	50.890	806.0000	NS
Num_of_seq	23.939	6.413	39.981	9.407	107.5000	0.000

*p values <0.01 were considered as significant*.

*NS means not significant*.

* *Bonferroni-corrected value*.

## Discussion

Several reports in the medical literature suggest the existence of a correlation and/or comorbidity between sleep disorders and headache linked to putative common pathophysiological substrates. In general, it has well-known that specific headache disorders, like paroxysmal hemicrania, cluster headache, and hypnic headache may be related to the rapid eye movement sleep (REM) or to obstructive sleep apnea syndrome (OSAS) (4). The details of the relationship mutual relationship between headache and sleep regulation are not still clearly understood, but it is known that sleep may be related to the occurrence of some headache syndromes while headache could cause or sustain various degrees of sleep disturbance (i.e., parasomnias, sleep disordered breathing, sleep-wake transition disorders). To date, in pediatric populations few studies seem to indicate a suggestive association between headaches and sleep disturbances, including primary snoring, obstructive sleep apnea, and NREM parasomnias although these data are mainly derived from questionnaire-based studies (8, 26–30).

On the other hand, clinical, and experimental data indicate that the thalamus may be considered as the key structure for migraine pathophysiology. EEG studies have shown that interictal migraine have low thalamo/thalamocortical transmission associated with low brainstem activation (31). In this picture, we could explain the low arousal index during REM sleep reported in children affected by MoA respect of healthy controls.

Moreover, some reports have showed that children affected by migraine may exhibit disrupted sleep architecture, such as abnormalities in total sleep time (TST) and sleep latency (SOL) compared with healthy control subjects (30). Conversely, the previous PSG study by Vendrame et al. (13) showed an important alteration/disruption in sleep in children affected by migraine and chronic migraine linked to the presence of sleep-disordered breathing, shortened TST, and high SOL, even if no healthy controls were used for comparisons.

About these alterations in TST and SOL, the Authors suggested that because some children may find relief from migraine attacks with daytime naps (or the sleep could be useful to stop the attacks), the attacks occurred during the daytime may impact the normal sleep-wake cycle (32). The severity and frequency of headache attacks may negatively affect sleep architecture provoking sleep disruptions and REM sleep percentages, as confirmed in adult subjects with migraine (33). Moreover, in adults, the reduction in REM sleep and number of arousals during REM was reported during the night preceding the migraine (34), and in this perspective a shorter sleep latency during the night before a migraine attack was observed also in children, suggesting a sort of decreasing in cortical activation the night before the onset of headache (35, 36).

Our findings seem to partially confirm some of the results reported previously such as the reduction in TST and SPT, but not in SOL and stages percentages, which in our sample, were not significantly different from healthy controls. As for the sleep disruption, our results seem to confirm the observation that children with migraine tend to show a higher rate of awakenings per hour respect of controls.

As for the NREM sleep instability analysis, the main finding of our study was the reduction in CAP rate percentage and also in N1 and N3. In our population, the CAP A1 representation was reduced in the total number and index, but with a prolonged duration than controls, and the CAP A2 and CAP A3 higher in the total number, and the CAP A2 with a longer duration in MoA vs. C. Our results are substantially in line with the data found in the study conducted in 2016 by Nayak et al. (16) on a sample of adults with MoA. In their findings the overall CAP rate, the number of CAP cycles and phase B duration was lower among migraineurs while the total phase A and phase 1 duration were increased.

Moreover, our findings confirm the reduction in CAP rate evidenced by Della Marca et al. in adults with frequent MoA (37). From this point of view, the reduction in oscillatory components during sleep in our sample could be reflecting a general hypoactivity of the arousal systems. Each of these systems has ascending projections to the cortex (which stimulate cortical activation and induce fast EEG activity) and descending projections to the spinal cord (which stimulate motor activation and induce high EMG activity) (38) and are located within the brainstem, the thalamus, the hypothalamus, and the basal forebrain (39). These areas could be considered actually as the generators of the migraine attacks (40, 41).

In our sample the CAP reduction involved prevalently the A1 phases subtypes, less so the high-frequency EEG arousals. One main role of CAP A1 fluctuations is to buffer the effect of perturbations occurring during NREM sleep (37). It can therefore be speculated that the reduction of CAP expresses a reduced efficacy of such mechanisms of processing of incoming inputs during sleep in migraine. Finally, we have to consider that to the best our knowledge, this is the first attempt to evaluate NREM instability and CAP parameters in children affected by migraine without aura compared with a control group.

In conclusion, the reduction of arousability and lower NREM sleep instability seem to be associated with MoA in children. These findings may have clinical implications. However, further studies are needed for a better comprehension of the pathophysiological mechanisms underlying the link between migraine and NREM sleep and to investigate possible consequent clinical implications and preventive treatments.

## Ethics Statement

The investigation was carried out in accordance with the principles of the Declaration of Helsinki (18). The Departmental Ethics Committee approved the study. Ethics committee protocol and approval was not considered as necessary, because the evaluation done is part of the clinical routine normally performed for children and adolescents in our Unit referred for migraine without aura.

## Author Contributions

MR, RM, FO, DS, FP, IB, GM, DI, and MC: substantial contributions to the conception or design of the work, or the acquisition, analysis, or interpretation of data for the work and agreement to be accountable for all aspects of the work in ensuring that questions related to the accuracy or integrity of any part of the work are appropriately investigated and resolved. MR, RM, FO, DS, FP, IB, GM, BG, ME, FS, GD, CL, MS, VR, PM, DI, and MC: drafting the work or revising it critically for important intellectual content and final approval of the version to be published. All authors read and approved the final manuscript.

### Conflict of Interest Statement

The authors declare that the research was conducted in the absence of any commercial or financial relationships that could be construed as a potential conflict of interest.
